# Examining levels, distribution and correlates of health insurance coverage in Kenya

**DOI:** 10.1111/tmi.12912

**Published:** 2017-07-05

**Authors:** Jacob S. Kazungu, Edwine W. Barasa

**Affiliations:** ^1^ Health Economics Research Unit KEMRI Wellcome Trust Research Programme Nairobi Kenya; ^2^ Nuffield Department of Medicine Oxford University Oxford UK

**Keywords:** health financing, health insurance, inequalities, Kenya, financement de la santé, assurance santé, inégalités, Kenya

## Abstract

**Objective:**

To examine the levels, inequalities and factors associated with health insurance coverage in Kenya.

**Methods:**

We analysed secondary data from the Kenya Demographic and Health Survey (KDHS) conducted in 2009 and 2014. We examined the level of health insurance coverage overall, and by type, using an asset index to categorise households into five socio‐economic quintiles with quintile 5 (Q5) being the richest and quintile 1 (Q1) being the poorest. The high–low ratio (Q5/Q1 ratio), concentration curve and concentration index (CIX) were employed to assess inequalities in health insurance coverage, and logistic regression to examine correlates of health insurance coverage.

**Results:**

Overall health insurance coverage increased from 8.17% to 19.59% between 2009 and 2014. There was high inequality in overall health insurance coverage, even though this inequality decreased between 2009 (Q5/Q1 ratio of 31.21, CIX = 0.61, 95% CI 0.52–0.0.71) and 2014 (Q5/Q1 ratio 12.34, CIX = 0.49, 95% CI 0.45–0.52). Individuals that were older, employed in the formal sector; married, exposed to media; and male, belonged to a small household, had a chronic disease and belonged to rich households, had increased odds of health insurance coverage.

**Conclusion:**

Health insurance coverage in Kenya remains low and is characterised by significant inequality. In a context where over 80% of the population is in the informal sector, and close to 50% live below the national poverty line, achieving high and equitable coverage levels with contributory and voluntary health insurance mechanism is problematic. Kenya should consider a universal, tax‐funded mechanism that ensures revenues are equitably and efficiently collected, and everyone (including the poor and those in the informal sector) is covered.

## Abstract (fr)

**Objectif:**

Examiner les niveaux, les inégalités et les facteurs associés à la couverture de l'assurance santé au Kenya.

**Méthodes:**

Nous avons analysé les données secondaires de l'Enquête Démographique et de Santé du Kenya menée en 2009 et 2014. Nous avons examiné le niveau de couverture de l'assurance santé en général et par type en utilisant un indice d'actifs pour classer les ménages dans 5 quintiles socioéconomiques, le quintile 5 (Q5) étant le plus riche et le quintile 1 (Q1) le plus pauvre. Le ratio élevé/faible (ratio Q5/Q1), la courbe de concentration et l'indice de concentration (CIX) ont été utilisés pour évaluer les inégalités dans la couverture de l'assurance santé et la régression logistique pour examiner les corrélats de la couverture de l'assurance santé.

**Résultats:**

La couverture globale de l'assurance santé est passée de 8,17% à 19,59% entre 2009 et 2014. Il y avait une forte inégalité dans la couverture générale de l'assurance santé, bien que cette inégalité ait diminué entre 2009 (Q5/Q1: 31,21 ; CIX = 0,61 ; IC95%: 0,52‐0,71) et 2014 (Q5/Q1: 12,34 ; CIX = 0,49 ; IC95%: 0,45‐0,52). Les personnes plus âgées, employées dans le secteur formel, mariées, exposées aux médias, de sexe masculin, appartenant à un petit ménage, souffrant d'une maladie chronique et appartenant à des ménages riches, avaient une probabilité accrue de couverture de l'assurance santé.

**Conclusion:**

La couverture d'assurance santé au Kenya reste faible et se caractérise par une inégalité importante. Dans un contexte où plus de 80% de la population est dans le secteur informel et près de 50% vivent en dessous du seuil national de pauvreté, l'atteinte de niveaux de couverture élevés et équitables de l'assurance santé avec un mécanisme contributif et volontaire est problématique. Le Kenya devrait envisager un mécanisme universel financé par l'impôt qui garantit que les impôts soient collectés équitablement et efficacement et que tous les individus (y compris les pauvres et ceux du secteur informel) sont couverts.

## Abstract (es)

**Objetivo:**

Examinar los niveles, las inequidades y factores asociados con la cobertura del seguro sanitario en Kenia.

**Métodos:**

Hemos analizado datos secundarios del Censo Demográfico y Sanitarios de Kenia (CDSK) realizado en el 2009 y 2014. Examinamos el nivel de cobertura del seguro sanitario, y por tipo, utilizando un índice de activos para categorizar los hogares en cinco quintiles socioeconómicos, en el que el quinto quintil (5Q) era el más rico y el primer quintil (1Q) el más pobre. La razón alto‐bajo (razón Q5/Q1), la curva de concentración y el índice de concentración (InC) se emplearon para evaluar las inequidades en la cobertura del seguro sanitario, y mediante una regresión logística se examinaron los correlatos de cobertura de los seguros sanitarios.

**Resultados:**

La cobertura general del seguro sanitario aumentó del 8.17% al 19.59% entre el 2009 y 2014. Había una alta inequidad en la cobertura total, aunque esta inequidad disminuyó entre el 2009 (ratio Q5/Q1 de 31.21, InC=0.61, IC 95% 0.52‐0.0.71) y el 2014 (ratio Q5/Q1 12.34, InC=0.49, IC 95% 0.45‐0.52). Los individuos que eran mayores, empleados en el sector formal, casados, expuestos a los medios, y pertenecían a hogares ricos, tenían una mayor probabilidad de tener cobertura de seguro sanitario.

**Conclusión:**

La cobertura del seguro sanitario en Kenia continúa siendo baja y está caracterizada por una inequidad significativa. En un contexto en el que más del 80% de la población está en el sector informal y cerca del 50% vive por debajo de la línea nacional de pobreza, el logro de unos niveles de cobertura altos y equitativos con un mecanismo contributivo y voluntario de seguro de salud es problemático. Kenia debería considerar un mecanismo universal y pagado por impuestos que asegure que las ganancias sean equitativas, eficientemente recogidas y que todos (incluidos los pobres y aquellos en el sector informal) estén cubiertos.

## Introduction

In 2005, WHO member states adopted a resolution to help member countries transform their health financing systems to achieve Universal Health Coverage (UHC) 1. UHC – defined as the provision of needed, and good quality health services to the entire population, without the risk of financial ruin 2, 3 – has received global support as recently enshrined in the Sustainable Development Goals (SDGs) 3.8 4. To achieve this goal, most low‐ and middle‐income countries (LMIC) are increasingly prioritising UHC and reforming their health systems to achieve it. One of these interventions used to achieve UHC is social health insurance schemes 5, 6.

Kenya has made a commitment to achieve UHC by 2030 7. To attain this, the government has undertaken a number of health reforms over the past years. For instance, in 2013 it abolished user fees in public primary healthcare facilities (health centres and dispensaries) and introduced a free maternity services policy in all public healthcare facilities 8. The Kenyan government also expanded the benefit package offered by the National Hospital Insurance Fund (NHIF) from an inpatient only package, to include outpatient services, and introduced a health insurance subsidy programme, whereby poor households are identified and given 100% subsidy on NHIF membership 9. The NHIF provides health insurance cover to both individuals in the formal and informal sector. Formal sector individuals pay an income‐rated monthly premium that is deducted automatically from the their salaries and remitted to the the NHIF by their employer. Formal sector premiums vary from KES 150 (USD 1.5) for the lowest income bracket (monthly salary of less than KES 6000 (USD 60)) to KES 1700 (USD 17) for the highest income bracket (monthly salary of above KES 100 000 (USD 1000)). Informal sector individuals pay a voluntary monthly flate rate premium of KES 500 (USD 5) 10, 11.

Kenya has a mixed health financing system with various sources of funding 12. Kenya's health sector is financed through tax revenues (31% of total health expenditure (THE) in 2012/13), donor funds (25% of THE), health insurance contributions (about 13% of THE) and out‐of‐pocket payments (27% of THE) 13. The high level of out‐of‐pocket payments means that financial risk protection is inadequate. The incidence of catastrophic healthcare expenditure in Kenya is estimated to be 4.52%, with 453 470 individuals pushed into poverty annually due to out‐of‐pocket healthcare payments 14.

A key policy decision that the Kenyan government has adopted is to expand voluntary, contributory health insurance as one of the strategies to achieve UHC 15. In addition to the NHIF, health insurance in Kenya is provided by private health insurance, employer‐provided health insurance, and community‐based and microhealth insurance 16. A qualitative assessment of factors influencing health insurance enrolement in Kenya revealed that even though there is a willingness to enrol, there are barriers such as lack of knowledge of health insurance enrolment options and procedures 17. Affordability of premium payments was also identified as a key barrier 17. Further, a willingness and ability to pay study reported that informal sector individuals were only willing to pay a maximum of KES 300 (USD 3) monthly premium, rather than the current NHIF monthly premium for these segments of the population (500 (USD 5)) 18. In addition to a concern for the average population coverage, a key concern for scaling up pre‐payment health financing mechanisms is the distribution of coverage (equity). Also, monitoring of these schemes is essential to attaining UHC. Against a background of Kenya's policy preference for health insurance, this study aimed to examine the levels, inequalities and correlates of health insurance coverage in Kenya.

## Methods

### Study setting

Kenya is a lower‐ to middle‐income country, ranked number 145 in the 2015 global Human Development Index ranks 19 and with an estimated population of 46.1 million in 2015 20; 65% of the population reside in rural areas, and the country has an estimated poverty rate of 45.9% 21. It adopted a devolved system of government in 2010 with the establishment of 47 county governments with key responsibilities in the provision and financing of health.

### Study design and data sets

We analysed secondary data from two rounds of the Kenya Demographic and Health Survey (KDHS) (2009 and 2014). The 2009 KDHS included a total of 400 primary sampling units that were used to select 3256 men and 8444 women aged 15 to 49 years 22. The 2014 KDHS included a total of 1612 primary sampling units that were used to select 12 014 men and 31 079 women aged 15 to 49 years 23. Both surveys used a nationally representative two‐stage cluster sampling design with stratification for rural and urban residence. DHS data sets are available in recode files. We utilised the female's individual recode (IR) and males recode (MR) for this analysis.

KDHS surveys are designed to collect an array of information about households and individuals, and among the information collected in some countries is health insurance. DHS survey interviewers obtain health insurance information by asking respondents whether they are covered by any form of insurance (response is ‘Yes’ or ‘No’). In some countries, including Kenya, respondents who are covered by any health insurance are also asked to state the specific types of insurance they are covered by (responses are ‘social health insurance’, ‘private insurance’, ‘community‐based’, ‘pre‐payment scheme’ and ‘other’).

### Data analysis

Before analysis, we restricted the age category of males to only those between 15 and 49 years for comparability between genders. We then combined the male and female data sets into a single data set for each of the KDHS surveys. In this analysis, we only included individuals with complete responses to our outcome variable (coverage by any form of insurance) resulting in a total of 11 690 (8435 women and 3255 men) in the 2009 KDHS and 26 743 (14 733 women and 12 010 men) in the 2014 KDHS. For modelling for the correlates of health insurance coverage in Kenya, only the 2014 KDHS data set was used because it was the most recent data set.

We conducted descriptive analysis to examine health insurance coverage – with any insurance and by specific health insurance – by the socio‐demographic factors identified in the literature 24, 25, 26, 27, 28. This included respondent age category, employment, gender, sex of the household head, residence, marital status, exposure to mass media, household size, education and socio‐economic status. To determine whether an individual's health status would determine health insurance coverage, we used the presence or absence of a chronic disease (hypertension/diabetes) as a proxy measure.

To identify factors associated with health insurance coverage, we first performed bivariate analysis using Pearson's chi‐square test (*X* ^*2*^). All factors found significant at *P*‐value<0.05 were incorporated into the multivariable logistic regression model. Prior to fitting the model, we assessed for potential multicollinearity using the Pearson's R correlation coefficient (*r* ≥ 0.8) 29. Our dependent variable in this analysis was coverage with health insurance (*No or Yes*). We examined the distribution of this variable in relation to a range of independent variables that have been suggested in literature to predict health insurance coverage specifically: respondent age category (*15–24, 25–34 and 35–49*); employment status (*unemployed, informally employed and formally employed*); sex (*female or male*); sex of the household head (*female or male*); residence (*urban or rural*); marital status (*not currently married or currently* married); exposure to radio, television or newspaper media (*not at all, less than once a week or at least once a week*); household size (*1–3, 4–5 and >5*); education (*no education, primary education, secondary education and tertiary/higher education*); wealth quintile (*poorest, poorer, middle, richer and richest*); *and presence or absence of* hypertension or diabetes (*no or yes)*. For the descriptive and correlates analysis, adjustments were made for the complex study design by accounting for the clustering and stratification survey design 30.

To assess inequality in health insurance coverage, we (i) computed the high‐to‐low ratio, (ii) developed concentration curves and (iii) computed the concentration index (CIX). The high‐to‐low ratio (Q5/Q1) is computed by dividing the level of health insurance coverage in the highest quintile, by the level of health insurance coverage in the lowest quintile. Given that it only compares individuals from the highest quintile (Q5) to those from the lowest quintile (Q1), and excludes the middle three quintiles (Q2, Q3 and Q4), this inequality measure is not generalisable to the whole population. We therefore calculated the concentration index to assess the existence, direction and magnitude of inequalities in health insurance coverage by wealth quintile 31. CIX is defined as twice the area between the concentration curve and the line of equality. A concentration curve is a plot of the cumulative percentage of the health variable–health insurance (*y*‐axis) against the cumulative percentage of the population ranked by socio‐economic status, from poorest to richest (*x*‐axis). A concentration index of zero denotes equality, while a negative (positive) concentration index denotes a pro‐poor (pro‐rich) distribution of the health variable 32. Data analysis was performed in STATA version 14.2 (Stata Corp, Lake way Drive, College Station, TX, USA).

## Results

### Health insurance coverage

Table 1 shows the distribution of the study sample characteristics for the 2009 and 2014 KDHS surveys. In both surveys, a majority of respondents were of age 15–24 (41.69% [95% CI 40.05–43.35] and 37.75% [95% CI 36.85–38.66]) years and were employed in the informal sector (41.38% [95% CI 39.38–43.42] and 62.72% [95% CI 61.54–63.88]). There was a considerable decrease in formal employment from 25.01% [95% CI 22.98–27.16] in the 2009 KDHS to only 11.19% [95% CI 10.40–12.03] in the 2014 KDHS. Exposure to media at least once a week increased between the same periods, rising from 41.26% [95% CI 38.74–43.83] to 83.24% [95% CI 82.23–84.21].

**Table 1:** Distribution of sample by socio‐demographic factors in the 2009 and 2014 KDHS Surveys

	2009 KDHS		2014 KDHS	
	(Weighted value) Total *n* = 11690		(Weighted value) Total *n* = 26 743	
	Total number (*n*)	% [95% CI]	Total Number (*n*)	% [95% CI]
Age category				
15–24	4874	41.69 [40.05–43.35]	10 096	37.75 [36.85–38.66]
25–34	3604	30.83 [29.11–32.61]	8999	33.65 [32.74–34.58]
35–49	3212	27.48 [26.41–28.57]	7648	28.60 [27.79–29.41]
Employment status				
Not employed	3929	33.61 [31.84–35.42]	6979	26.10 [25.15–27.07]
Informal employment	4837	41.38 [39.38–43.42]	16 772	62.72 [61.54–63.88]
Formal employment	2924	25.01 [22.98–27.16]	2992	11.19 [10.40–12.03]
Respondent gender				
Female	8435	72.16 [71.04–73.23]	14 656	54.80 [54.01–55.59]
Male	3254	27.84 [26.77–28.96	12 087	45.20 [44.41–45.99]
Sex of the household head				
Female	3638	31.12 [28.96–33.37]	7663	28.65 [27.50–29.84]
Male	8052	68.88 [66.63–71.04]	19 080	71.35 [70.16–72.50]
Place of residence				
Urban	3010	25.75 [20.60–31.67]	11 253	42.08 [40.09–44.09]
Rural	8680	74.25 [68.33–79.40]	15 490	57.92 [55.91–59.91]
Marital status				
Not married	5176	44.28 [42.63–45.94]	11 910	44.54 [43.44–45.64]
Married	6514	55.72 [54.06–57.37]	14 833	55.46 [54.36–56.56]
Exposure to media				
Not at all	4122	35.26 [32.96–37.63]	2143	8.01 [7.43–8.64]
Less than once a week	2745	23.48 [21.91–25.13]	2338	8.74 [8.04–9.50]
At least once a week	4823	41.26 [38.74–43.83]	22 262	83.24 [82.23–84.21]
Household size				
1–3	2896	24.78 [21.92–27.87]	8332	31.16 [29.73–32.62]
4–5	3735	31.95 [30.09–33.88]	8913	33.33 [32.15–34.53]
>5	5059	43.27 [39.65–46.97]	9498	35.52 [34.05–37.01]
Level of education				
No education	861	7.37 [5.89–9.19]	1363	5.09 [4.58–5.66]
Primary education	6480	55.43 [52.46–58.36]	13 168	49.24 [47.84–50.64]
Secondary	3413	29.20 [26.32–32.25]	8958	33.50 [32.41–34.60]
Higher	936	8.00 [6.60–9.67]	3254	12.17 [11.01–13.43]
Household socio‐economic status				
Poorest	1847	15.80 [13.52–18.37]	3936	14.72 [13.55–15.97]
Poorer	2057	17.60 [15.42–20.00]	4745	17.74 [16.73–18.80]
Middle	2185	18.69 [16.65–20.92]	5240	19.59 [18.44–20.81]
Richer	2457	21.02 18.50–23.78]	6084	22.75 [21.29–24.28]
Richest	3144	26.89 [21.98–32.44]	6737	25.19 [23.27–27.22]
Having a chronic disease				
No	‐	‐	24 902	93.12 [92.70–93.51]
Yes	‐	‐	1841	6.88 [6.49–7.30]

The 2009 KDHS did not include questions on having a chronic disease (hypertension or diabetes) and alcohol consumption.

### Trends in health insurance coverage

**Figure 1:**
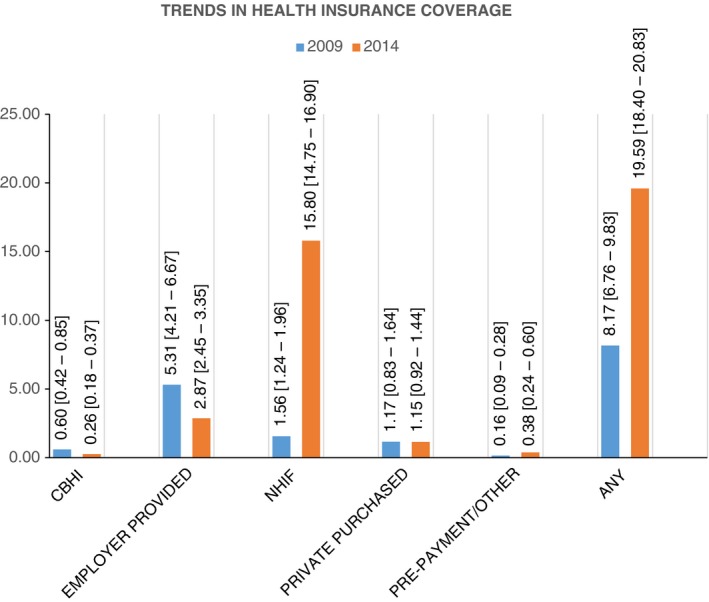
Trends in health insurance coverage in Kenya.

Overall, health insurance coverage in Kenya increased from 8.17% [95% CI 6.76 ‐ 9.83] to 19.59% [95% CI 18.40–20.83], between 2009 and 2014. Figure 1 shows the levels of health insurance coverage by type over the two survey periods. Coverage by the NHIF increased almost eightfold (from 1.56% [95% CI 1.24–1.96] to 15.80% [95% CI 14.75–16.90]) between 2009 and 2014. However, coverage with the community‐based, employer‐provided and private health insurance decreased marginally between the two survey rounds.

Table 2 presents the results of the distribution of health insurance coverage by a range of selected variables. Health insurance coverage in men improved more (from 11.30% [95% CI 9.23–13.77] to 21.35% [95% CI 19.87–22.91]) compared to women (from 6.96% [95% CI 5.64–8.55] to 18.13% [95% CI 16.90–19.43]).

**Table 2:** Health insurance coverage and its distribution by selected independent variables in the 2009 and 2014 KDHS surveys

Covered by health insurance	2009 KDHS			2014 KDHS		
	(Weighted value) Total *n* = 11 690		% insured [95% CI]c	(Weighted value) Total *n* = 26 743		% insured [95% CI]
	Total number	*n* insured		Total number	*n* insured	
Age category						
15–24	4874	189	3.88 [2.78–5.38]	10 096	1077	10.67 [9.58–11.87]
25–34	3604	379	10.50 [8.40–13.06]	8999	2209	24.55 [22.73–26.45]
35–49	3212	387	12.06 [10.01–14.45]	7648	1952	25.52 [23.79–27.33
Employment status						
Not employed	3929	138	3.52 [2.61–4.73]	6979	667	9.56 [8.40–10.85]
Informal employment	4837	255	5.28 [3.78–7.32]	16 772	3009	17.94 [16.72–19.23]
Formal employment	2924	561	19.19 [16.18–22.61]	2992	1561	52.20 [48.93–55.45]
Respondent gender						
Female	8435	587	6.96 [5.64–8.55]	14 656	2657	18.13 [16.90–19.43]
Male	3254	368	11.30 [9.23–13.77]	12 087	2581	21.35 [19.87–22.91]
Sex of the household head						
Female	3638	249	6.86 [5.39–8.68]	7663	1213	15.83 [14.44–17.32]
Male	8052	705	8.76 [7.21–10.60]	19 080	4025	21.10 [19.75–22.51]
Place of residence						
Urban	3010	562	18.68 [15.45–22.40]	11 253	3124	27.76 [25.64–30.00]
Rural	8680	393	4.52 [3.74–5.46]	15 490	2113	13.64 [12.48–14.90]
Marital status						
Not married	5176	263	5.09 [3.93–6.57]	11 910	1550	13.01 [11.76–14.38]
Married	6514	691	10.61 [8.82–12.73]	14 833	3688	24.86 [23.37–26.42]
Exposure to media						
Not at all	4122	306	7.43 [5.75–9.54]	2143	65	3.02 [2.24–4.06]
Less than once a week	2745	150	5.47 [4.25–7.03]	2338	212	9.08 [7.35–11.18]
At least once a week	4823	498	10.33 [8.20–12.95]	22 262	4961	22.28 [20.96–23.67]
Household size						
1–3	2896	391	13.49 [10.27–17.51]	8332	2129	25.55 [23.57–27.64]
4–5	3735	289	7.73 [6.31–9.43]	8913	1931	21.66 [19.88–23.57]
>5	5059	276	5.45 [4.43–6.68]	9498	1178	12.40 [11.15–13.78]
Level of education						
No education	861	3	0.29 [0.12–0.68]	1363	37	2.74 [1.94–3.85]
Primary education	6480	177	2.73 [1.93–3.85]	13 168	1409	10.70 [9.72–11.77]
Secondary education	3413	399	11.69 [9.31–14.58]	8958	1995	22.27 [20.58 ‐ 24.06]
Tertiary/Higher	936	376	40.22 [35.51–45.12]	3254	1796	55.21 [51.29–59.07]
Household socio‐economic status						
Poorest	1847	12	0.66 [0.34–1.32	3936	124	3.16 [2.46–4.04]
Poorer	2057	25	1.20 [0.70–2.06]	4745	378	7.97 [6.89–9.20]
Middle	2185	73	3.33 [2.35–4.70]	5240	721	13.75 [12.07–15.63]
Richer	2457	195	7.94 [6.42–9.79]	6084	1395	22.93 [21.08–24.88]
Richest	3144	650	20.68 [17.58–24.16	6737	2620	38.89 [36.20–41.64]
Having a chronic disease						
No	‐	‐	‐	24 902	4713	18.93 [17.78–20.14]
Yes	‐	‐	‐	1841	524	28.49 [25.08–32.15]

2009 KDHS did not include questions on having a chronic disease (hypertension or diabetes).

Across both survey years, health insurance coverage tended to increase with age, exposure to media, the level of education, socio‐economic status, formal employment status, urban residence and among the married. However, health insurance coverage decreased with increase in household size. In the 2014 KDHS, health insurance coverage also increased among those with a chronic disease (diabetes/hypertension) compared to their counterparts without a chronic disease.

### Inequalities in health insurance coverage

Our findings indicate the existence of pro‐rich income‐related inequalities in health insurance coverage in Kenya. Individuals from the wealthiest quintile were more than 12 times more likely to be covered with any type of health insurance compared to the poorest quintile in the 2014 KDHS with an overall pro‐rich distribution of insurance coverage (CIX = 0.49 [95% CI 0.45–0.52]) (Table 3). This was an improvement on the 2009 KDHS where the high‐to‐low ratio was 31 and the concentration index was 0.61 [95% CI 0.52–0.71]). This pattern is repeated for all forms of health insurance. The gap between the wealthiest and the poorest is greatest when the employer‐provided and private health insurance (PHI) are considered (high‐to‐low ratio of 26 and 20, respectively, in 2014).

**Table 3:** Socio‐economic distribution of health insurance coverage, and concentration index (CIX) by type of health insurance in the 2009 and 2014 KDHS surveys

	2009		2014	
	High‐to‐low ratio	Concentration index (CIX)	High‐to‐low ratio	Concentration index (CIX)
Any	31.21	0.61 (0.52–0.71)	12.34	0.49 (0.45–0.52)
CBHI	5.30	0.37 (0.13–0.61)	3.64	0.29 (0.09–0.50)
Employer‐provided HI	41.38	0.61 (0.49–0.73)	26.36	0.57 (0.45–0.69)
NHIF	73.40	0.53 (0.39–0.67)	12.27	0.44 (0.40–0.48)
Private purchased HI	58.67	0.74 (0.50–0.98)	20.18	0.63 (0.46–0.81)
Pre‐payment/Other scheme	‐	0.64 (0.25–1.04)	3.71	0.31 (0.09–0.53)

‐Denotes an infinite value due to zero coverage in the poorest quintile (Q1).

**Figure 2:**
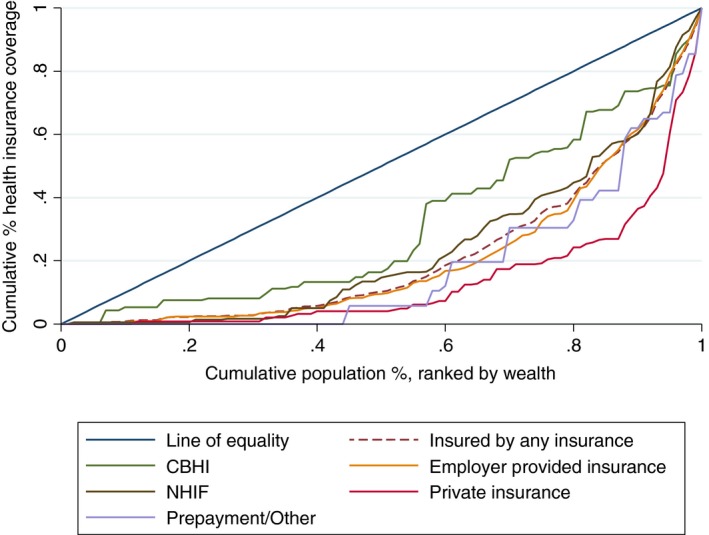
Concentration curve for specific health insurance coverage in the 2009 KDHS.

Figure 2 presents the concentration curves for health insurance coverage with specific health insurance types in the 2009 KDHS. Although all forms were pro‐rich, the concentration curve for the CBHI was closer to the line of equality than that for PHI indicating lower inequalities in the CBHI than PHI.

**Figure 3:**
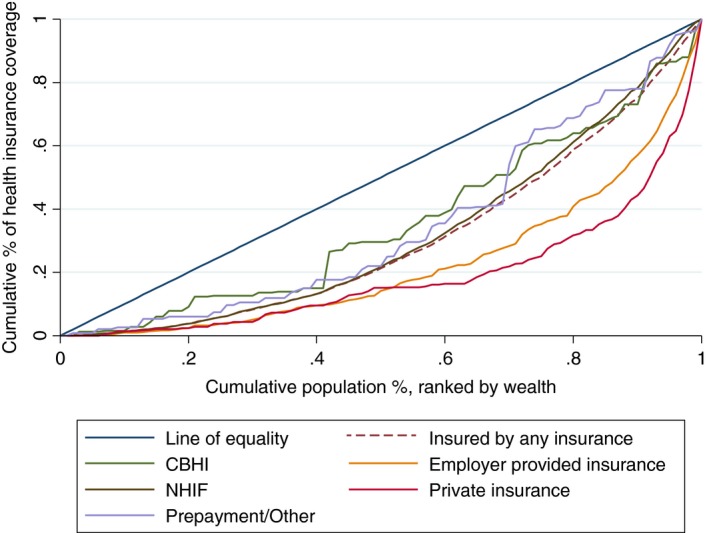
Concentration curve for specific health insurance coverage in the 2014 KDHS.

Figure 3 presents the concentration curves for health insurance coverage with specific health insurance in the 2014 KDHS. The curves show a similar presentation to the ones in 2009 KDHS, indicating the continuity of pro‐rich inequalities.

**Figure 4:**
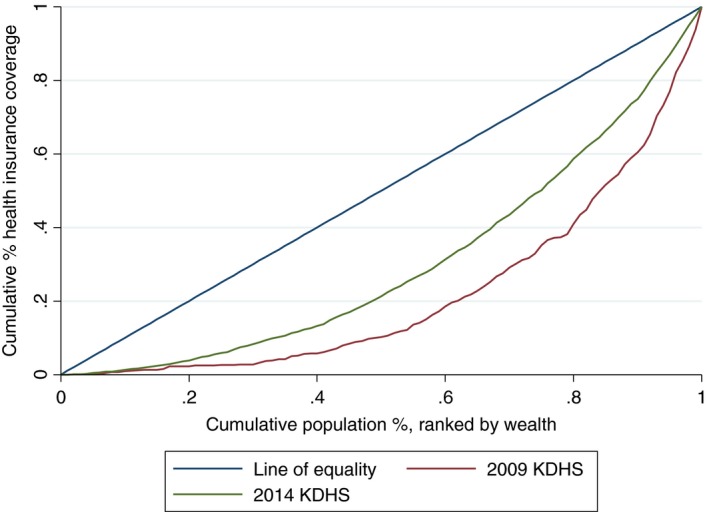
Concentration curve for health insurance coverage in Kenya, 2009 and 2014.

Figure 4 presents the concentration curves for overall health insurance coverage in 2009 and 2014 KDHS surveys. Both curves are below the line of equality indicating that health insurance coverage remains pro‐rich. However, dominance test indicated that the two curves are significantly apart. Therefore, health insurance coverage in 2014 is more equitable than in 2009 KDHS.

### Correlates of health insurance coverage

Table 4 shows the results from the bivariate and multivariable logistic regression for determining the correlates of health insurance coverage. Findings from the multivariable logistic regression indicated that individuals between the age of 35 and 49 were almost twice as likely to be insured as those in the 15–29 years bracket (AOR = 1.92; 95% CI 1.65–2.25). While individuals with any form of employment had higher odds of being insured than unemployed individuals, the odds were almost 3 times higher for those employed in the formal sector (AOR = 2.65; 95% CI 2.19–3.28). Men had no greater odds of being insured compared to women even when they were heads of households. Unexpectedly, individuals from urban areas had a 19% decreased odds of being insured than their counterparts from rural areas (AOR = 0.81; 95% CI 0.68–0.97). Compared to unmarried individuals, married people had significantly greater odds of being covered with health insurance (AOR = 1.97; 95% CI 1.73–2.25). Exposure to media also contributed to a positive change in the probability of health insurance coverage with exposure to media at least once a week having 2.22 times more odds of coverage compared to those not exposed to media at all (AOR = 2.22; 95% CI 1.58–3.11). Household size had a negative relationship with health insurance coverage. Individuals from households with one to three members had 30% greater odds of coverage than their counterparts from households with more than five members (AOR = 1.30 95% CI 1.10–1.54). The odds of coverage increased with the level of education with odds of coverage of those with tertiary or higher education being 9.41 times those without education (AOR = 9.41; 95% CI 36.24–14.19). Those from the wealthiest quintile were 7.34 times more likely of being insured compared to their counterparts in the poorest quintile (AOR = 7.34; 95% CI 5.29–10.18). Despite having a borderline significance, individuals with a chronic disease (hypertension/diabetes) had 22% (AOR 1.22 95% CI 1.00–1.48) greater odds of coverage.

**Table 4:** Odds ratios (OR), adjusted odds ratios (AOR), 95% confidence intervals and *P*‐values for predictors of health insurance coverage: using the 2014 KDHS

	Bivariate analysis			Multivariable analysis		
	Odds ratio (OR)	95% confidence interval	*P*‐value	Adjusted odds ratio (AOR)	95% confidence interval	*P*‐value
Age category (Reference 15–29)						
20–34	2.72	2.38–3.12	0.00	1.54	1.30–1.83	0.00
35–49	2.87	2.55–3.22	0.00	1.92	1.65–2.25	0.00
Employment status (Reference not employed)						
Informal employment	2.07	1.81–2.37	0.00	1.33	1.13–1.57	0.01
Formal employment	10.34	8.72–12.25	0.00	2.68	2.19–3.28	0.00
Respondent gender (Reference female)						
Male	1.23	1.13–1.33	0.00	1.11	1.00–1.24	0.06
Sex of the household head (Reference female)						
Male	1.42	1.28–1.58	0.00	0.95	0.84–1.08	0.416
Place of residence (Reference rural)						
Urban	2.43	2.10–2.82	0.00	0.81	0.68–0.97	0.02
Marital status (Reference not married)						
Married	2.21	1.98–2.47	0.00	1.97	1.73–2.25	0.00
Exposure to media (Not at all)						
Less than once a week	3.21	2.17–4.75	0.00	1.83	1.23–2.73	0.00
At least once a week	9.21	6.72–12.64	0.00	2.22	1.58–3.11	0.00
Household size (Reference >5)						
4–5	1.95	1.68–2.27	0.00	1.21	1.04–1.41	0.01
1–3	2.42	2.07–2.84	0.00	1.30	1.10–1.54	0.00
Level of education (Reference no education)						
Primary education	4.26	2.96–6.12	0.00	2.07	1.41–3.04	0.00
Secondary education	10.18	7.06–14.68	0.00	4.05	2.73–6.00	0.00
Tertiary/Higher	43.81	29.76–64.49	0.00	9.41	6.24–14.19	0.00
Socio‐economic status (Reference poorest)						
Poorer	2.66	1.99–3.53	0.00	1.83	1.35–2.48	0.00
Middle	4.89	3.67–6.52	0.00	2.97	2.17–4.05	0.00
Richer	9.12	6.92–12.02	0.00	4.57	3.37–6.19	0.00
Richest	19.51	14.73–25.84	0.00	7.34	5.29–10.18	0.00
Having a chronic disease (Reference no)						
Yes	1.71	1.45–2.01	0.00	1.22	1.00–1.48	0.05

## Discussion

This study presents an analysis of the level, inequalities and correlates of health insurance coverage in Kenya using data from two rounds (2009 and 2014) of the nationally representative KDHS. Our findings showed an increase in overall health insurance coverage. However, despite this increase, a significant proportion (80%) of the population in 2014 remains uninsured, underscoring the slow progress in extending coverage to the whole population. Several factors may contribute to this. First, Kenya has a large (83%) and growing informal sector for whom coverage is voluntary 33. International evidence suggests that it is problematic to achieve high levels of coverage among the informal sector population using a voluntary, contributory mechanism 17, 34. Among others, challenges include unpredictable and irregular incomes and logistical difficulties in regularly collecting premium contributions from individuals in the informal sector 35. Second, close to 50% of Kenyans live below the national poverty line 36. This means that they cannot afford to pay insurance premiums and implies that a voluntary, contributory approach is unlikely to achieve any meaningful level of coverage.

Among those that are covered, our findings reveal significant inequalities in health insurance coverage. While this is expected for private health insurance, where contributions are based on ability to pay, it is instructive that forms of health insurance that are ideally expected to cater for the poor, such as public health insurance (NHIF), and community‐based health insurance, are also associated with significant inequalities. This again emphasises the fact that voluntary and contributory health insurance mechanisms that are typically based on ability to pay are predisposed to inequalities, and are perhaps not appropriate in settings with high informality and poverty.

Our findings offer insights on factors that are associated with health insurance coverage. Older age, employment, being married, exposure to media, smaller household size, higher education, higher socio‐economic status, the presence of a chronic disease, increased the odds of having health insurance. These findings are consistent with evidence from other settings on the determinants of health insurance coverage 17, 24, 26, 27, 37, 38. While the bivariate analysis showed that individuals living in urban areas had increased odds of having health insurance compared to those living in the rural areas, this effect was not only attenuated but in fact reversed by the effect of socio‐economic status. Most of the poorer individuals in our sample resided in rural areas compared to urban areas.

The association between health insurance coverage and employment, and also with socio‐economic status further emphasises the potential for inequality of contributory, voluntary health insurance. For instance, richer individuals and those in the formal sector have an increased odds of having health insurance coverage compared to poorer individuals, and those in the informal sector, respectively. Finally, the fact that individuals with chronic diseases (diabetes or hypertension) were significantly more likely to be insured than their counterparts without chronic diseases has implications for the viability of health insurance schemes as this may represent a form of adverse selection.

### Limitations

Our findings should be interpreted with consideration to some limitations. First, the analysis is based on a cross‐sectional survey data set. We therefore could not be able to measure causality and settled for associations. Second, the data set is based on data collected in 2014, and hence, the picture could be different in the present day. Third, our study was not able to quantify the relationship between breadth and depth of insurance coverage due to lack of data on the benefits packages covered by the health insurance in Kenya. Future studies should explore this relationship to understand whether coverage would change with changes in the benefits package.

## Conclusion

Against a background of Kenya's policy decision to pursue a voluntary, contributory health insurance mechanism, we offer one key recommendation; Kenya should reconsider its decision and instead adopt a predominantly tax funded mechanism to extend coverage with a pre‐payment mechanism to its population. While considering fiscal constraints, Kenya should tax fund 100% subsidies for the poor through the public insurer, NHIF. The poor may never be able to pay premiums under a contributory and voluntary mechanism because they lack the ability to pay. Further, Kenya should provide a partial health insurance subsidy, through the NHIF to individuals in the informal sector in the short term. Registering and more importantly retaining informal sector individuals to the NHIF will continue to be a challenge under a voluntary and contributory mechanism. In the long term, Kenya should implement a universal mechanism that ensures that everyone is covered by the NHIF, funded by tax funds.

## References

[1] Organization WH . Sustainable health financing, universal coverage and social health insurance. World Health Assembly Resolution. 2005;58.

[2] Organization WH . WORLD HEALTH REPORT (The): Health Systems Financing: the path to universal Coverage World Health Organization; 2010. 10.2471/BLT.10.078741 PMC2878164 20539847

[3] Obare V , Brolan CE , Hill PS . Indicators for universal health coverage: can Kenya comply with the proposed post‐2015 monitoring recommendations? Int J Equity Health 2014: 13: 123. 25532714 10.1186/s12939-014-0123-1 PMC4296682

[4] Assembly UG . Transforming Our World: The 2030 Agenda for Sustainable Development. New York: United Nations, 2015.

[5] Lagomarsino G , Garabrant A , Adyas A , Muga R , Otoo N . Moving towards universal health coverage: health insurance reforms in nine developing countries in Africa and Asia. Lancet 2012: 380: 933–943. 22959390 10.1016/S0140-6736(12)61147-7

[6] McIntyre D . Learning from Experience: Health care financing in low‐and middle‐income countries. Research: Global Forum for Health; 2007.

[7] Vision K . 2030–The Popular Version. Government of Kenya, Nairobi. 2007.

[8] Maina TKD . An evaluation of the abolition of user fees at primary healthcare facilities in Kenya. Washington, DC; 2015.

[9] Mwaura RN , Barasa E , Ramana G , Coarasa J , Rogo K . The Path to Universal Health Coverage in Kenya. The World Bank; 2015.

[10] National Hospital Insurance Fund (Standard and Special Contributions) Regulations. Legal Notice No.14., (2015).

[11] Munge K , Mulupi S , Chuma J . A critical analysis of the purchasing arrangements in Kenya: the case of the National Hospital Insurance Fund, Private and Community‐based health insurance. 2015. 10.15171/ijhpm.2017.81 PMC5890069 29524953

[12] Chuma J , Okungu V . Viewing the Kenyan health system through an equity lens: implications for universal coverage. Int J Equity Health 2011: 10: 22. 21612669 10.1186/1475-9276-10-22 PMC3129586

[13] Kenya MoH‐Go . Kenya National Health Accounts 2012/2013. Nairobi; 2015.

[14] Barasa EW , Maina T , Ravishankar N . Assessing the impoverishing effects, and factors associated with the incidence of catastrophic health care payments in Kenya. Int J Equity Health 2017: 16: 31. 28166779 10.1186/s12939-017-0526-x PMC5294805

[15] Ministry of Medical Services . Sessional Paper No. 7 of 2012 on The Policy on Universal Health Care Coverage in Kenya. In: Services MoM, editor. Nairobi, Kenya: Ministry of Medical Services; 2012.

[16] Kenya MoH . 2013 Kenya household expenditure and utilization survey. Nairobi; 2014.

[17] Mathauer I , Schmidt JO , Wenyaa M . Extending social health insurance to the informal sector in Kenya. An assessment of factors affecting demand. Int J Health Plann Manage 2008: 23: 51–68. 18050152 10.1002/hpm.914

[18] Fund NHI . Willingness and ability to pay for the National Hospital Insurance Fund (NHIF) insurance package for the informal sector in Kenya. 2016.

[19] Jahan S , Jespersen E , Mukherjee S *et al* Human development report 2015: Work for human development. UNDP: New York, NY, USA. 2015.

[20] World Bank . 2016 [cited 10/03/2017]. Available from: http://www.worldbank.org/en/country/kenya/overview.

[21] Ministry of Health‐ Kenya . 2013 Kenya Household Health Expenditure and Utilisation Survey. Nairobi; 2014.

[22] KNBS I . Macro: Kenya Demographic and Health Survey 2008‐09. Calverton, MD: Kenya National Bureau of Statistics and ICF Macro. 2010; 430.

[23] Demographic K . Health Survey 2014: key indicators. Kenya National Bureau of Statistics (KNBS) and ICF Macro. 2014.

[24] Bourne PA , Kerr‐Campbell MD . Determinants of self‐rated private health insurance coverage in Jamaica. Health 2010: 2: 541.

[25] Kimani JK , Ettarh R , Warren C , Bellows B . Determinants of health insurance ownership among women in Kenya: evidence from the 2008‐09 Kenya demographic and health survey. Int J Equity Health 2014: 13: 27. 24678655 10.1186/1475-9276-13-27 PMC3973618

[26] Kiplagat IJ . Determinants of health insurance choice in Kenya: University of Nairobi, Kenya; 2011.

[27] Kirigia JM , Sambo LG , Nganda B , Mwabu GM , Chatora R , Mwase T . Determinants of health insurance ownership among South African women. BMC Health Serv Res 2005: 5: 17. 15733326 10.1186/1472-6963-5-17 PMC553985

[28] Onwujekwe O , Okereke E , Onoka C , Uzochukwu B , Kirigia J , Petu A . Willingness to pay for community‐based health insurance in Nigeria: do economic status and place of residence matter? Health Policy Plan 2010: 25: 155–161. 20156920 10.1093/heapol/czp046

[29] Katz MH . Multivariable Analysis: A Practical Guide for Clinicians. New York: Cambridge University Press, 2006.

[30] Lumley T . Analysis of complex survey samples. J Stat Softw 2004: 9: 1–19.

[31] Wagstaff A , Van Doorslaer E , Paci P . Equity in the finance and delivery of health care: some tentative cross‐country comparisons. Oxford Rev Econ Pol 1989: 5: 89–112.

[32] O'donnell O , Van DE , Wagstaff A , Lindelow M . Analyzing Health Equity using Household Survey Data. Washington, DC: World Bank, 2008.

[33] Kenya National Bureau of Statistics . Economic Survey 2016. Nairobi: Kenya National Bureau of Statistics, 2016.

[34] Wagstaff A , van Doorslaer E , van der Burg H *et al* Equity in the finance of health care: some further international comparisons. J Health Econ 1999: 18: 263–290. 10537896 10.1016/s0167-6296(98)00044-7

[35] Resilient and Responsive Health Systems . Covering the Informal Sector: Report from a workshop on expanding access to health services and financial protection for people outside the formal employment sector. London: RESYST; 2014.

[36] Kenya National Bureau of Statistics . Kenya Integrated Household Budget Survey, Revised. Nairobi: Kenya National Bureau of Statistics; 2005.

[37] Kimani DN , Muthaka DI , Manda DK . Healthcare financing through health insurance in Kenya: The shift to a national social health insurance fund: Kenya Institute for Public Policy Research and Analysis; 2004.

[38] Chuma J , Kirigia D , Mulupi S . Community perceptions of health insurance and their preferred design features: implications for the design of universal health coverage reforms in Kenya. 2013. 10.1186/1472-6963-13-474 PMC3842821 24219335

